# News and Notes

**Published:** 1904-06

**Authors:** 


### NEWS AND NOTES.

A Resident Physician is wanted for the Tabernacle Infirmary. One having some acquaintance with bacteriology and pathology preferred. Address or apply to Dr. R. R. Kime, English-American Building, Atlanta, Ga.

---

Dr. J. B. Morgan has been elected President of the Board of Health of Augusta.

---

Two hundred and thirty-six ambulances were shipped from Indianapolis last month for Japan.

---

The mortality in Zion City, the colony of Dowieites, is said to be 35 in 1,000. There are other things worse than doctors and drug stores.

---

The Pennsylvania Society for the Prevention of Tuberculosis has determined to begin an active campaign for the prevention of the spread of tuberculosis as well as other infectious diseases.

---

Subscriptions for the proposed Wesley Memorial Hospital were taken up among the Methodist churches throughout the State on April 24, and sums to the amount of $50,000 were subscribed.

---

The Harvard Medical School experts who are going to the Philippines to study smallpox germs have a method in their apparent mania. They will carry on their operations in some of the distant islands of the archipelago, not because smallpox prevails there, but because monkeys are many and antivivisectionists few.

DAILY papers inform us that the Russian admiral in charge in the far east is suffering from the effects of a severe nervous depression. One of our readers asks, "Is it not possible that his disease is in reality chorea?"—*Exchange.*

---

BUILDING COMMISSIONER WILLIAMS, of Chicago, has notified those in charge of eight of the smaller hospitals in that city that they must receive no new patients in their institutions until certain specified building changes are made.—*Exchange.*

---

THE County Detention Hospital at Ann Arbor, Mich., will soon be ready for the reception of contagious diseases, the county having arranged to raise the $30,000 necessary for the building, and the university having promised to meet the running expenses.

---

THE American Laryngological, Rhinological and Otological Society will hold its tenth annual meeting in Chicago, Ill., May 30, 31, and June 1, 1904, under the presidency of Dr. Norval H. Pierce. Wendell C. Phillips, secretary, 49 West Forty-seventh street.

---

THE Senatus Academicus of the University of Edinburgh has awarded the Cameron prize in practical therapeutics to Prof. Niles R. Finsen, M.D,, of Copenhagen, in recognition of his pioneer work in connection with the application of light rays to the treatment of disease.

---

IT is reported from Vienna, says the *Medical Record,* that Professor Gussenbauer has relieved a cancerous stricture of the esophagus by means of radium. A rubber capsule containing sixty milligrammes of radium was attached to the end of an esophageal tube and passed down to the seat of the stricture.

After a certain time, which was not definitely stated in the cabled report, the stricture yielded and the tube passed into the stomach.

---

THE city council of the French city of Rouen has adopted the plan of offering a prize of two dollars to the first person who notifies the authorities of a case of typhoid fever. A sum of four dollars will likewise be paid to the family if they agree to follow the directions of the board of health in regard to disinfection, etc. When the city council adopted the above typhoid bounty plan the only two dissenting votes were those of the two physicians who serve as members of the council.

---

THE food test at the Department of Agriculture, which has been going on for several months past, to test the effect of salicylic acid on human health, has brought out some queer results. The general fact is established, says *American Medicine*, that salicylic acid seriously injures health. One man, however, has reason to rejoice in the salicylic acid treatment. It has cured his rheumatism. The experiments have been abandoned to save the subjects, but will be resumed when the men have recovered somewhat from the effects of the past two months' diet on prepared foods into which salicylic acid entered as a preservative. Dr. Wylie says that he expected this result. This experiment has turned out just as did that with foods preserved with borax. That made all the men sick.

---

THE nuisance of beggars in India is intensified by the number of leprous and diseased cripples who haunt the thoroughfares of most towns. In Bombay it is claimed that they stand in rows endeavoring to excite the compassion of passers-by. The sight is not only offensive but revolting, and there is probably danger in allowing so many lepers to move about and to live in the most densely populated quarters of the town. The very worst cases and the most contagious are the most in evidence. In Calcutta this traffic in sores is also a serious and constant evil. There is a growing opinion that the time has come for these pitiable mortals to be prevented from turning their misfortunes to account in earning a livelihood to the nuisance and danger of the public, and that adequate provision will have to be made to keep them out of the way.—*Lancet.*

---

CHILD labor, says *American Medicine,* is declining in New York State. Not only has there been a large decrease in the total number of children receiving permits to work, but there has been also almost a total elimination of the perjury resorted to by parents under the old law to get children into factories and stores. The establishment of systematic co-operation between the authorities who enforce the law and the authorities who investigate and, if necessary, relieve the poverty conditions so often alleged as the cause of child labor, is announced in the report of the Child Labor Committee just issued. The new law has been in operation since October 1, 1903. During October, November and December certificates were issued in New York City to 2,922 children, or 67 per cent. of all who applied, whereas during the same months of the preceding year certificates were issued to 4,353 children, or 80 per cent. of all who applied. The stricter requirements which have caused this change are a minimum age, a minimum amount of schooling, and proof that the child has been observing the compulsory school law.

